# Wheat proteomics: proteome modulation and abiotic stress acclimation

**DOI:** 10.3389/fpls.2014.00684

**Published:** 2014-12-08

**Authors:** Setsuko Komatsu, Abu H. M. Kamal, Zahed Hossain

**Affiliations:** ^1^National Institute of Crop Science, National Agriculture and Food Research OrganizationTsukuba, Japan; ^2^Plant Stress Biology Lab, Department of Botany, West Bengal State UniversityKolkata, India

**Keywords:** wheat, proteomics, abiotic stress, review

## Abstract

Cellular mechanisms of stress sensing and signaling represent the initial plant responses to adverse conditions. The development of high-throughput “Omics” techniques has initiated a new era of the study of plant molecular strategies for adapting to environmental changes. However, the elucidation of stress adaptation mechanisms in plants requires the accurate isolation and characterization of stress-responsive proteins. Because the functional part of the genome, namely the proteins and their post-translational modifications, are critical for plant stress responses, proteomic studies provide comprehensive information about the fine-tuning of cellular pathways that primarily involved in stress mitigation. This review summarizes the major proteomic findings related to alterations in the wheat proteomic profile in response to abiotic stresses. Moreover, the strengths and weaknesses of different sample preparation techniques, including subcellular protein extraction protocols, are discussed in detail. The continued development of proteomic approaches in combination with rapidly evolving bioinformatics tools and interactive databases will facilitate understanding of the plant mechanisms underlying stress tolerance.

## Introduction

Wheat is one of the major food crops worldwide, and the glutens and storage proteins in wheat grain are the single greatest source of protein in the human diet (Gill et al., 2004). In particular, the wheat species *Triticum aestivum* L. provides one-fifth of the total calories the world's population (Reynolds et al., 2010). To further improve wheat yields, it is necessary to develop varieties of wheat that can be managed using methods that preserve local environments and natural resources (Ribeiro et al., 2013). To meet this challenge, the integration of wheat genomics, transcriptomics, and proteomics with rapidly evolving bioinformatics tools and interactive databases is required.

The genome of common wheat is large (17 Gb) (Safár et al., 2010) and complex due to numerous polyploidy events that occurred between 8000 and 10,000 years ago (Gupta et al., 2008; Brenchley et al., 2012). The wheat genome is essentially comprised of the DNA of three different primitive species, which may explain the great capacity of wheat plants to adapt to various ecological conditions (Brenchley et al., 2012). The sequencing of the wheat genome is enabling a more effective and focused approach to the breeding of high-yielding varieties with increased tolerance to environmental stresses. The International Wheat Genome Sequencing Consortium recently published a chromosome-based draft sequence of the bread wheat genome (Brenchley et al., 2012), an accomplishment that is expected to facilitate the breeding of varieties that are tolerant to the biotic and abiotic stresses that cause yield losses. However, because knowledge of a genomic sequence alone does not indicate how a plant interacts with the environment, and not all open reading frames correspond to a functional gene (Ribeiro et al., 2013), proteomics approaches are critical for understanding plant mechanisms of stress tolerance.

The present review highlights the major proteomic findings in studies examining wheat acclimation responses to abiotic stresses. Moreover, the strengths and weaknesses of different sample preparation techniques, including subcellular protein extraction protocols, are discussed. The continued development and application of these proteomics techniques will provide new insights into the underlying mechanisms of stress tolerance in wheat.

## Sample preparation techniques

### Total protein extraction

Sample preparation is the most crucial aspect of proteomics analysis. The preparation of total proteins from plants is considerably more challenging compared to other organisms due to the abundance of plant proteases and other compounds such as polyphenol, polysaccharides, starch, lipids, and secondary metabolites, which interfere with protein detection by causing proteolytic breakdown, streaking and charge heterogeneity. Moreover, certain tissues contain highly abundant proteins that hamper the isolation, separation, visualization, and accurate identification of the complete proteome. For example, the presence of the extremely abundant photosynthetic CO_2_ fixation enzyme ribulose 1,5-bisphosphate carboxylase/oxygenase (RuBisCO) in leaves not only limits the dynamic resolution of low-abundance target proteins, but also impairs the detection of other proteins and affects the electrophoretic mobilities of neighboring protein species (Herman et al., 2003). Different fractionation techniques based upon the physiological or biochemical properties of RuBisCO have been used to reduce or remove this enzyme by polyethylene glycol and DTT from total leaf protein extracts (Kim et al., 2001; Cho et al., 2008; Widjaja et al., 2009). For example, an affinity column containing anti- RuBisCO large subunit antibody and protein A-Sepharose as a resin effectively eliminated RuBisCO from protein extracts of rice chloroplasts (Hashimoto and Komatsu, 2007). Krishnan and Natarajan (2009) developed a relatively fast and simple fractionation technique using 10 mM calcium and 10 mM phytate to precipitate 85% of the total RuBisCO from soluble protein extract of soybean leaf. These techniques can also be used for the preparation of wheat proteins less contaminated with RuBisCO.

In contrast, accurate quantitation of RuBisCO itself is another challenge for proteomics study of plant stress response. Capillary electrophoresis is often used as an effective method for recovery of RuBisCO from the crude plant extract. In this process RuBisCO extracted from plant leaves is first completely denatured into small and large subunits in presence of SDS. The SDS-RuBisCO complexes are then separated by using an uncoated fused-silica capillary filled with a replaceable polymer solution, with detection at 220 nm (Chen et al., 2000). However, presence of phenolic compounds in plant extract often limits the use of this method. Interestingly, addition of insoluble polyvinylpolypyrrolidone during leaf extraction was found to be effective in effective removal of phenols thus, allowing accurate quantitation of RuBisCO (Warren et al., 2000). Leech and Marrison (1996) exploited cyto-immunofluorescence technique for accurate quantitation of RuBisCO per chloroplast in wheat. In this process, PEG-embeded transverse leaf sections were first hybridized with RuBisCO antisera followed by a secondary antibody conjugated to fluorescein isothiocyanate (FITC). The fluorescence output was measured against the standard curve prepared through rocket immunoelectrophoresis. In this method, RuBisCO levels in the leaf tissue were quantified by measuring rocket peak heights and then comparing these with the known concentrations of purified wheat RuBisCO protein (Laurell, 1966).

**Figure 1:**
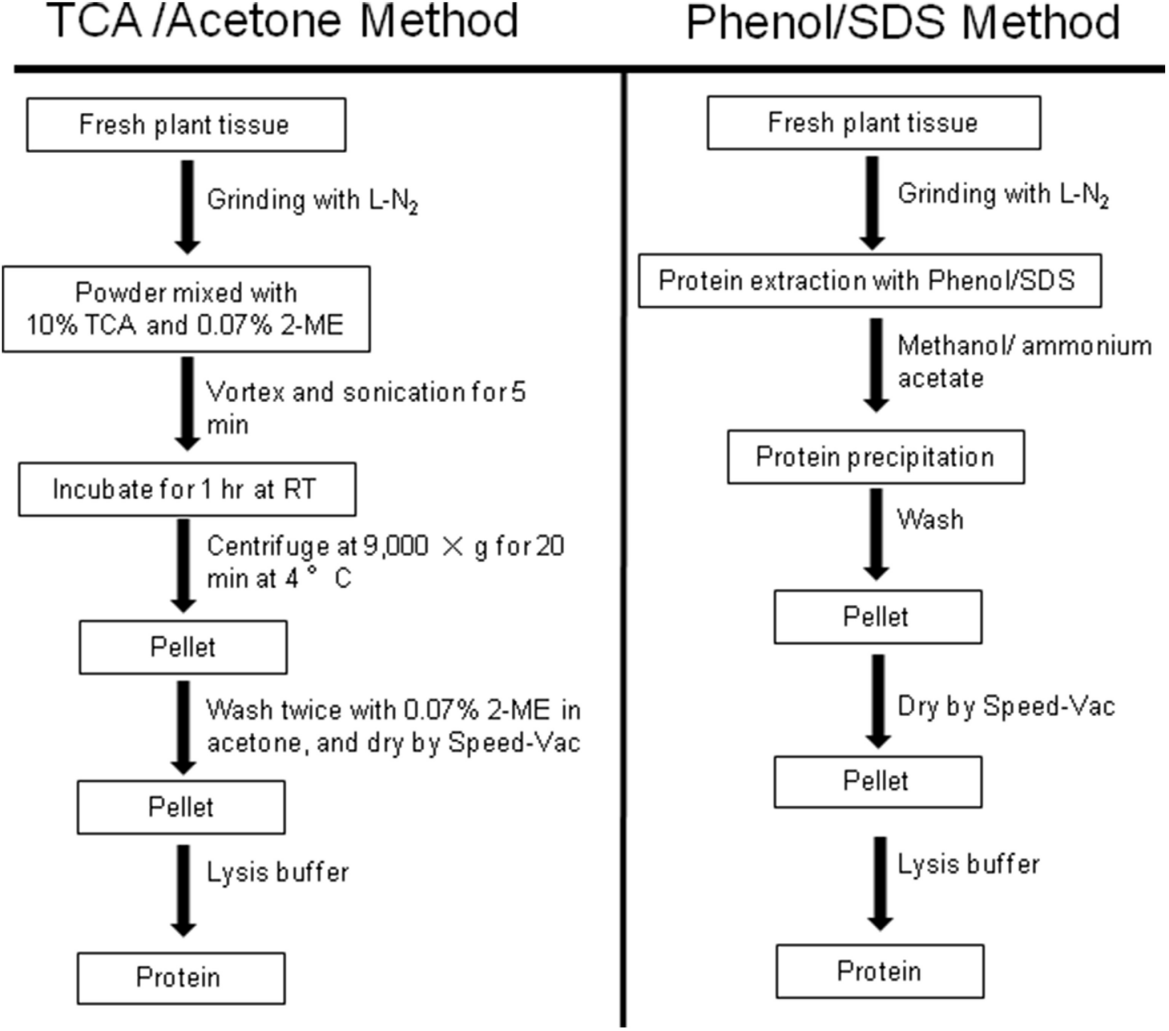
**Flowchart showing the steps of plant protein extraction**. For trichloroacetic acid (TCA)/acetone, fresh plant tissues are first ground to powder in liquid nitrogen. The powder is transferred to TCA and 2-mercaptoethanol (2-ME) in acetone, vortexed, and the resulting suspension is sonicated. After a 1-h incubation with vortexing, the sample is centrifuged and the obtained pellet is washed twice with 2-ME in acetone. The washed pellet is dried using a Speed-Vac concentrator, and resuspended in lysis buffer for analysis (Kim et al., 2010). For phenol/SDS extraction methods, dried powder of plant tissue is resuspended in phenol/SDS buffer, and the mixture is then vortexed thoroughly. After separating the phenol phase by centrifugation, the upper phenol phase is glycera to fresh tubes. At least 5 volumes of cold methanolic ammonium acetate is added to the phenol phase and the resulting mixture is stored at −20°C. Precipitated proteins are recovered by centrifugation and then washed twice each with cold methanolic ammonium acetate and cold acetone prior to analysis (Bancel et al., 2010).

Standardization of sample preparation protocols to optimize protein yields and overcome the physicochemical limitations inherent to most techniques (Table 1). The recently developed trichloroacetic acid/acetone precipitation method (Kim et al., 2010; Shin et al., 2011) has been shown in wheat to be highly reproducible compared to the other methods (Figure 1). Although the final protein pellet is occasionally resistant to solubilization in this method, protein contaminants and plant pigments are effectively removed under acidic and/or hydrophobic conditions, resulting in high-quality gels. Another popular approach for total protein preparation is phenol extraction, which has a strong capacity for the removal of contaminants (Figure 1) (Bancel et al., 2010). In this protocol, it is critical that the sample is kept at very low temperature during the extraction, and the phenolic phase must be carefully recovered after each centrifugation step. The efficient solubilization of extracted proteins is an important step in all proteomic sample preparation methods to achieve sufficient protein concentrations. Moreover, the selection of the most suitable protocol is dependent on protein abundance, molecular weight, charge, hydrophobicity, post-translational processing and modifications, and presence of inhibitory molecules. To date, however, no specific protocol has been developed that is effective for all protein extractions.

**Table 1:** **Summary of wheat proteome analyses examining plant responses to abiotic stresses and others**.

**Stress/Conditions**	**Treatment time and dose**	**Cultivar**	**Organ/Organelle**	**Proteomic technologies**	**Stress induced modulation of metabolic pathways**	**Differentially expressed protein classification**		**References**
						**Functions**	**Localization**	
Flooding	7 d	Bobwhite line SH 9826	Seminal root	2-DE, nano LC-MS/MS	Antioxidant defense	StrRes	–	Haque et al., 2011
Flooding	2 d	Shiroganekomugi	Root	2-DE, nano LC-MS/MS	Carbohydrate (glycolysis)	EnMet, ProtMet, SigTran, Tranp	Cell wall	Kong et al., 2010
Drought	100 d	Opata, Nesser	Root	iTRAQ	Energy metabolism, Replication, repair	EnStr, OxiRed, Trans,	Mem,Cyto, Cell wall, Mito, Nucl, Plast, Vacu	Alvarez et al., 2014
Drought	7 d	Ofanto	Leaf	2-DE, MALDI-TOF	Carbohydrate (glycolysis, gluconeogenesis)	PTR, StrRes, TCA, ROSsca, AAB, GG	–	Caruso et al., 2009
Drought	7 d	Katya, Sadovo, Zlatitza, Miziya	Leaf	SDS-PAGE, 2-DE	Energy (photosynthesis)	EnMet, EnvDevS	Chlo	Demirevska et al., 2009
Drought	9 d	Keumkang	Leaf	2-DE, MALDI-TOF/TOF	Energy (photosynthesis)	Photo	Chlo	Kamal et al., 2013a
Drought	10, 15, 20, and 25 d	Janz, Kauz	Seed	2-DE, MALDI-TOF	Carbohydrate metabolism	ROSsca, CarMet, SigTran	–	Jiang et al., 2012
Drought	14, 24 d	Kukri, Excalibur, RAC87	Leaf	iTRAQ	Energy (photosynthesis)	Photo, GG, ProtF, Tranp, EnStr	–	Ford et al., 2011
Drought	20% PEG	Hanxuan 10 and Ningchun 47	Leaf	nano LC-MS/MS	Antioxidant defense	DRM, SigTran, StrRes, ROSsca	–	Zhang et al., 2014
Heat and Drought	10 d	Vinjett	Kernel	2-DE, MALDI-TOF	Carbohydrate (glycolysis)	CarboMet, STP	–	Yang et al., 2011
High temperature	37°C d, 28°C N/10 d, 20 d	Butte 86	Endosperm	2-DE, QSTAR PULSAR-TOF	Carbohydrate metabolism	CarboMet, NitMet, ProtMet, StrRes, STP, SigTran, Tranp, Trans	–	Hurkman et al., 2009
Salt	150 mM NaCl/1 d, 2 d, 3 d	Keumkang	Leaf	2-DE, LTQ-FTICR-MS	Energy (photosynthesis)	Photo, StrRes	Chlo	Kamal et al., 2012a
Salt	1.0, 1.5, 2.0, and 2.5% NaCl in HS/2 d	Zhengmai 9023	Leaf	2D-DIGE/ Q-TOF-MS	Carbohydrate metabolism	CarMet, ProtF, Tranp, ROS, ATP	–	Gao et al., 2011
Salt	200 mM	Wyalkatchem, Janz	Shoot	2-DE, LC-MS/MS	–	–	Mito	Jacoby et al., 2010
Aluminum	250 μM/2 d, 3 d	Atlas-66, Fredrick	Root	SDS-PGE, Immunoblot	Signaling pathway	Oxi	–	Delisle et al., 2001
Aluminum	100, 150 μM/5 d	Keumkang	Root	2-DE, LTQ-FTICR-MS	Energy (glycolysis)	Gly, Tranp, SigTran, StrRes, EnMet	–	Oh et al., 2014
Copper	100 μM/3 d	Yumai 34	Root, Leaf	2-DE, HPLC-Chip/ ESI-Q-TOF/MS/MS	Energy (photosynthesis), antioxidant defense	StrRes, SigTran, ProtMet, CarMet, Photo, EnMet	–	Li et al., 2013
Protein profiling	20 d	Keumkang	Leaf	SDS-PAGE, LTQ-FTICR	Energy (photosynthesis)	COB, DevPro, DRM, ProtF, ProtMet, StrRes, Tranp, Trans	Chlo	Kamal et al., 2012b
Protein profiling	Mature seed	Wild type (AA, BB, DD genome)	Seed	SDS-PAGE, nano LC-MS/MS	Carbohydrate metabolism	StrRes, EnMet, ProtS, CGD, COD, ProtF, SigTran, STP, Tranp	–	Kim et al., 2010
Cadmium	10, 100, and 200 μM	Yangmai 15	Leaf	IPG, MALDI-TOF	Energy (photosynthesis)	Oxi, ProtMet, Photo	–	Wang et al., 2011
Cadmium	0.5 mM/L	Yangmai 13	Leaf	IPG, MALDI-TOF	Antioxidant defense	ROSsca	–	Ge et al., 2009

*AAB, amino acid biosynthesis; ATP, ATP synthase; CarMet, carbohydrate metabolism; CGD, cell growth and division; COB, cell organization, COD, cellular organization and development; DevPro, developmental process; DRM, DNA and RNA metabolism; EnMet, energy metabolism; EnStr, environmental stress; EnvDevS, environmental and developmental signals; GG, glycolysis and gluconeogenesis; Gly, glycolysis; NitMet, nitrogen metabolism; Oxi, oxidative stress; OxiRed, oxidation-reduction process; Photo, photosynthesis; ProtF, protein folding; ProtMet, protein metabolism; ProtS, protein synthesis; PTR, post-transcriptional regulation; ROSsca, ROS scavenging; SigTran, signal transduction; STP, storage proteins; StrRes, stress response; TCA, calvin cycle; Tranp, transport; Trans, translation, Chlo, chloroplast; Mem, membrane; Cyto, cytoplasm Nucl, nucleus; Mito, mitochondria; Plast, plastid; Vacu, vacuole; HS, hoagland solution*.

### Subcellular protein extraction

#### Cell wall purification

The cell wall is an important subcellular organelle for the modulation of stress signals and exhibits changes in protein abundance in response to environmental stress. Cell wall proteins have been extracted and purified from wheat roots under flooding stress (Feiz et al., 2006). The purification of plant cell walls is hampered by a number of technical difficulties such as contamination from other organelles. Thus, characterization of the cell wall proteome remains challenging and requires a combination of various treatment and analytical approaches (Watson et al., 2004). For *Arabidopsis*, Jamet et al. (2008) described a protocol for purifying soluble and weakly bound cell wall proteins with only low levels of contamination by intracellular proteins, thus providing a more acurate description of protein functions in the apoplast (Jamet et al., 2008). However, extraction methods developed for *Arabidopsis* may not be useful for other species. A procedure for extracting and purifying cell wall proteins was adopted for wheat seedling roots, and the purity of the cell wall protein extract was confirmed by measuring the activity of glucose-6-phosphate dehydrogenase (Kong et al., 2010). Structural differences in the cell walls between species could have consequences to cell susceptibility to rupture by infiltration techniques, whereas compositional differences in the matrix, such as the content of homogalacturonic acid, might require the use of different extraction buffers for more effective protein release from the matrix. This implies that for the first proteomic studies of a plant species, the level of contamination of the cell wall extract must be monitored carefully and the extraction protocol adjusted accordingly to maximize its content of cell wall proteins (Komatsu and Yanagawa, 2013).

#### Chloroplast and mitochondria isolation

Photosynthesis in plant, algae, and cyanobacteria uses the energy from sunlight to covert carbon dioxide and water into chemical energy. Chloroplast isolation and purification from various organs or cell types are critical steps for the profiling of the chloroplast proteome. Intact chloroplasts of wheat have been isolated and purified from fully developed leaves on Percoll gradients (D'Amici et al., 2009; Kamal et al., 2012b). On the other hand, mitochondria have been considered the most attractive targets for subcellular proteomics because of the wide range of functions that they perform in cells. According to Jacoby et al. (2010), mitochondria were isolated from shoots in wheat using differential centrifugation followed by polyvinylpyrrolidone-40 gradient. Most of the proteins in the chloroplast envelop membrane and mitochondria membrane is hydrophobic in nature, so that it is technically difficult to analyze them by gel-based proteomic technique. This problem can, however, be resolved by an approach involving protein extraction with organic solvents together with a gel-free proteomic technique.

#### Plasma membrane purification

In studies involving membrane proteomics, designing suitable method of extraction and identification of entire set of hydrophobic proteins remains a challenge (Komatsu et al., 2009). Isolation of microsomal membrane fraction through “differential centrifugation” is the most common procedure in comprehensive plasma membrane proteomic analysis. The microsomal fraction, collected as the last pellet of ultracentrifuge protocol is the fraction of interest as it usually contains desired membranes such as endoplasmic reticulum, plasma membranes, Golgi apparatus, vacuolar membranes, and different types of endosomal vesicles (Abas and Luschnig, 2010). Using similar ultracentrifuge protocol, Basu et al. (1994) successfully fractionated microsomal membrane proteins from aluminum stressed wheat roots. Ahsan et al. (2012) also effectively isolated root microsomal proteins to examine Cd uptake and translocation in two contrasting Cd-accumulating soybean cultivars. In contrast to the above mentioned conventional ultracentrifugation method, Abas and Luschnig (2010) standardized a method for isolating microsomal-type membranes from *Arabidopsis* using lower relative centrifugal force of a microcentrifuge. This protocol incorporates specific manipulation of sample density throughout the procedure, with minimal preclearance, minimal volumes of extraction buffer, and minimal sedimentation path length. It also avoids losses during “preclearance” step, thereby ensuring maximal membrane yield.

## Flooding-induced changes in wheat proteome composition

### Root proteomics of early stage wheat under flooding stress

Cell wall proteins are important both for maintenance of cell structure and for responses to abiotic and biotic stresses; especially flooding stress induced the cell wall loosening in the early stage of plant growth (Komatsu and Yanagawa, 2013). However, neither wheat cell wall proteomics nor any cell wall response to flooding stress identified by proteomics in plants has been previously studied. A procedure for extracting and purifying plant cell wall proteins as adopted for wheat seedling roots, and the purity of the cell wall protein extract was assessed by measuring the activity of glucose-6-phosphate dehydrogenase. To identify flooding-stress responsive proteins in the wheat cell wall, gel-based and mass spectrometry (MS)-based proteomic techniques were applied. A total of 18 and 15 proteins were shown to accumulate in response to flooding by the former and latter proteomic techniques, respectively (Table 1). Among the accumulated proteins detected at lower levels in response to flooding, most were related to the glycolysis pathway and cell wall structure and modification (Kong et al., 2010). In contrast, the cell wall proteins of highest abundance after flooding treatment belonged to the category of defense and disease-response proteins (Kong et al., 2010). In addition to the identified accumulated proteins, a number of root proteins, including methionine synthase, β-1,3-glucanase, β-galactosidase, and β-glucosidase, were decreased in wheat in response to flooding. Among them, methionine synthase plays a pivotal role in methionine synthesis, which is essential for plant cell growth (Huang et al., 2006). The other enzymes, namely β-1,3-glucanases, β-galactosidase, and β-glucosidase belong to the glycosyl hydrolase family of proteins, are involved in the modification of cell wall polysaccharides (Lee et al., 2007). The decrease of these proteins suggests that wheat seedlings respond to flooding stress by restricting cell growth to avoid energy consumption. Thus, by coordinating methionine assimilation and cell wall hydrolysis, cell wall proteins appear to play critical roles in flooding stress acclimation in wheat (Kong et al., 2010).

### Proteomics of seminal roots of wheat under flooding stress

Seminal roots of seedlings play an important role in nutrient acquisition. The length and number of seminal roots may be particularly important in the acquisition of immobile nutrients by increasing soil exploration as well as inter-root competition (Zhu et al., 2006). Plant roots are particularly sensitive to oxygen deficit caused by waterlogging because they must absorb oxygen from the soil. In wheat, the seminal root system is the first organ that experiences severe waterlogging (Oyanagi, 2008). Hence, a rapid and well-organized response to the environment is essential in root meristems in low-oxygen soil (Haque et al., 2011). In Table 1, to examine these responses, proteins were extracted from wheat roots under flooding stress, separated by two-dimensional polyacrylamide gel electrophoresis (2-DE), and analyzed by nano-liquid chromatography (LC)-MS/MS. Sixteen proteins in 10 protein spots were significantly changed in the seminal roots of wheat in response to flooding stress (Haque et al., 2011).

Among the wheat root proteins that are increased during flooding stress, ADP-ribosylation factor 1 is known to play a role in vesicular trafficking and induction of phospholipase D activity (Memon, 2004). Vesicular trafficking drives directional root hair tip growth in *Arabidopsis* (Yoo et al., 2008) and induces the synthesis of matrix and cellulosic polysaccharides during cell wall construction (Lanubile et al., 1997). ADP-ribosylation factor 1 might be associated with alteration of the cell and cell wall structure during aerenchyma formation in roots under hypoxic conditions. The ubiquitin-conjugating enzyme spm2, which targets proteins for degradation in proteasomes via the ubiquitination reaction (Nandi et al., 2006), was also increased in the seminal roots of wheat under flooding conditions. Supermine modulates the expression of genes encoding redox components and is involved in protein folding, secretion, and degradation, and host defense. The increased activity of this enzyme in the seminal roots of wheat exposed to flooding might have an important role in protein degradation during cell degeneration in the process of aerenchyma formation in the root cortex (Haque et al., 2011).

Peroxidases are involved in lignification, suberization, auxin catabolism, wound healing, and defense against pathogen infection. The increase in peroxidaes might be associated with the higher lignin content in the cell wall of root cortex cells under flooding stress during aerenchyma formation (Erdmann et al., 1986), as well as host defense responses (Haque et al., 2011). Pathogenesis-related protein 1.2 has also been implicated as flooding-stress responsive proteins in the roots of wheat. The corresponding gene is specifically expressed in roots, and is up-regulated by rice blast fungus infection and various abiotic stresses, such as drought and salinity (Qu et al., 2006). The accumulation of this protein in the seminal roots of wheat suggests its involvement in adaptive responses to hypoxic conditions (Haque et al., 2011, 2014).

## Changes in wheat proteome composition under drought and salinity stresses

### Changes in wheat proteome composition under salt stress

Excess soil salinity is an ever-present threat to crop yields, particularly in arid and semi-arid zones. Extreme low temperature in winter wheat at either autumn seedling stage prior to verbalization or early spring crown stage can cause severe crop damage and reduce production (Xu et al., 2013). High temperatures during grain filling might be affected the dough properties and quality characteristics of wheat. Responses to high temperature have been related to changes in protein composition at both quantitative and qualitative levels (Majoul et al., 2003). Na^+^ and Cl^−^ ions are affected synergistically to salt toxicity in wheat to exert maximum damage to growth. The toxicity is expressed as a reduction in both plant growth and the photosynthetic apparatus (Martin and Koebner, 1995). High levels of soil C1^−^ can interfere with uptake of nitrate, leading to N starvation (Grattan and Grieve, 1992). Salinity, which is primarily due to the presence of excess Na^+^, is considered to be the single most widespread soil toxicity problem limiting global agricultural production, including wheat Excessive Na^+^ imparts both ionic and osmotic stresses to plant cells. Acclimation to salinity involves the synthesis of compatible solutes as well as adjustments in ion transport, including ion uptake, extrusion, and sequestration. This kind of proceedings ultimately lead to detoxification and a restitution of cellular homeostasis, allowing for protecting the plant under salinity condition (Chaves et al., 2009). Salinity stress were induced stomatal shutting, response inhibition due to decline descend activity, decreased RuBisCO levels, displacement of critical cations from endo-membranes structures, and swelling and disorganization of the grana lead to reduced photosynthesis (Peng et al., 2009). In addition, high salt concentrations (Na^+^) may directly affect stomatal conductance by reducing guard cell turgor and intercellular CO_2_ partial pressure (Kamal et al., 2012a).

**Figure 2:**
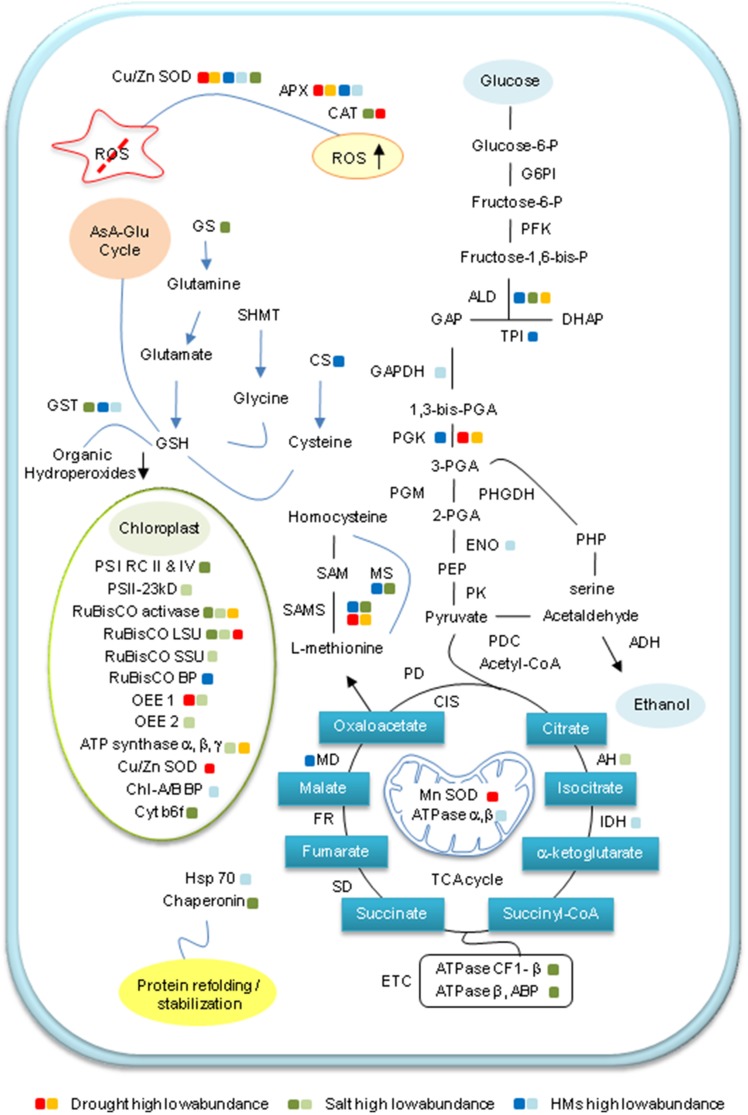
**Abiotic stress-induced changes in major metabolic pathways of wheat**. The proposed scheme is based on the results of proteomic studies examining changes in the wheat proteome in response to abiotic stress. High- and low-abundance proteins under the three different stress conditions (drought, salt, and heavy metals [HM]) are represented by dark- and light-shaded colored boxes, respectively. ADH, alcohol dehydrogenase; AH, aconitate hydratase; ALD, aldolase; APX, ascorbate peroxidase; AsA-Glu, ascorbate glutathione; CAT, catalase; CS, cysteine synthase; ENO, enolase; GAPDH, glyceraldehydes 3-phosphate dehydrogenase; G6PI, Glucose-6-phosphate isomerase; GS, glutamine synthetase; GSH, reduced glutathione; GST, glutathione-S-transferase; HM, heavy metal; Hsp, heat shock proteins; IDH, isocitrate dehydrogenase; LSU, large subunit; MD, malate dehydrogenase; MS, methionine synthase; OEE, oxygen-evolving enhancer protein; PFK, Phosphofructokinase; PGK, phosphoglycerate kinase; PHP, 3-phosphohydroxypyruvate; PK, pyruvate kinase; PS I, photosystem I; ROS, reactive oxygen species; RuBisCO, ribulose-1,5-bisphosphate carboxylase oxygenase; SAMS, S-adenosylmethionine synthetase; SAM, S-adenosylmethionine; SD, succinate dehydrogenase; SOD, superoxide dismutase; SSU, small subunit; and TPI, triose-phosphate isomerase.

In Table 1, a number of proteomic techniques have been used to identify salinity-induced changes in protein networks and thereby gain insight into wheat salinity stress responses (Wang et al., 2008; Peng et al., 2009; Gao et al., 2011). The impact of salt stress on protein expression patterns in wheat chloroplasts was recently monitored by 2-DE coupled with linear quadruple trap-Fourier transform ion cyclotron resonance (LTQ-FTICR) hybrid MS (Kamal et al., 2012a). Most differentially changed proteins exhibited higher abundances following an initial decrease on exposure to salt stress. This rapid change in protein abundance may represent a counterbalancing effect among the identified proteins. In addition, salinity stress significantly reduced the photosynthesis and transpiration rates, stomatal conductance, relative water content, and levels of Chl *a* and *b*, although proline levels were increased. In addition, photosystem I reaction center subunits II and IV, oxygen-evolving proteins (OEEs), and cytochrome b6–f (Cyt b6–f) complex decreased on exposure to salt stress (Figure 2).

RuBisCO is a stroma-localized protein and constitutes up to 50% of all chloroplast proteins. The expression of RuBisCO in wheat chloroplasts is strongly inhibited by salt treatment. In NaCl-treated rice, 19 different fragments of the RuBisCO large subunit have been detected, suggesting that it might be degraded during stress (Yan et al., 2006). Among differentially expressed chloroplast proteins in salt-stressed wheat seedlings, nucleoprotein might be concerned for chloroplast mRNAs processing (Baginsky and Gruissem, 2002) as well as light-induced activation of translation (Trebitsh and Danon, 2001) was identified. Salt stress treatments have paradoxical impacts on the thylakoid CF1-CF0 complex (ATP synthase). For instance, ATP synthase (α, β, γ, and ε) was modulated by salinity stress in wheat, and maize chloroplasts (Zörb et al., 2009), but changed in unevenly. The α and γ subunits generally increased at initial stage, whereas the ε subunit decreased over time. In contrast, the β subunit was changed dynamically under salt stress in chloroplast of wheat (Kamal et al., 2012a).

Proteome analysis of salt-stressed wheat chloroplasts revealed that five subunits of catalase (CAT), which is well-characterized antioxidant enzyme that protects cells from the toxic effects of hydrogen peroxide, displayed increased levels (Nishikawa et al., 2009). Superoxide dismutase (SOD) and CAT inactivate superoxide radicals (O^−^_2_) and H_2_O_2_, which are prevented the configuration of the most reactive form of reactive oxygen species (ROS), the hydroxyl radical (-OH). SOD dismutase the superoxide into oxygen and H_2_O_2_, while CAT converts H_2_O_2_ into water and oxygen. Because these enzymes are responsible for detoxifying ROS, monitoring their activities can be used indirectly to predict ROS production in plant cells (Yang and Poovaiah, 2002). However, the WoLF PSORT sub-cellular predictor indicates that all of the CAT subunits, with the exception of CAT3, are located in chloroplasts (Kamal et al., 2012a).

In addition to CAT, chlorophyll a-b binding proteins (CAB) were unevenly changed under salt stress in wheat chloroplasts (Kamal et al., 2012a). Tsugane et al. (1999) showed in *Arabidopsis* that expression of the light-harvesting complex, which is normally decreased at the mRNA level, but increased under salinity conditions. The up-regulation might be involved for changing the carbon flux in response resulting decreased the photosynthesis and osmotic adjustment (Li et al., 2011). Consistent with this speculation, the levels of the photosynthetic protein ribulose-bisphosphate carboxylase were decreased by salt stress in chloroplast of wheat, whereas a few proteins increased. Salt concentration and RuBisCo activity were changed reversely. Salt stress increases oxidation and decreases carboxylation activities of RuBisCO, and causes to decrease in severity of CO_2_ fixation (Sivakumar et al., 2000). RuBisCO activities were decreased by salinity may due to sensitivity of RuBisCO to Cl^−^ (Seemann and Critchley, 1985). The eukaryotic translation initiation factor complexes (eIF 3i, 5A-1/2, and 5A-3) were engaged in protein synthesis, which is stimulated the mRNA binding and methionyl-tRNAi to the 40S ribosome. eIF 5A-1/2, 5A-3, and eIF-3i were dynamically changed under salt stress in chloroplast of wheat (Kamal et al., 2012a). Thus, alteration of the salt concentration in the environment leads to increased or decreased translational activity in a cell or an organism depending on the nature of the stimulus (Burks et al., 2001).

In wheat, aldolases may therefore play a role in acclimating wheat seedlings to anaerobic conditions by reducing oxidative stress (Kamal et al., 2012a). The three isoforms of glyceraldehyde-3-phosphate dehydrogenase (GAPDH) identified to date, GAPA, GAPB, and GAPC, were decreased in wheat seedlings after 3 days of salt stress (Kamal et al., 2012a). In rice, the expression of mRNA for aldolases is enhanced by salt stress (Salekdeh and Komatsu, 2007). For example, fructose-bisphosphate aldolase, which catalyze the cleavage of fructose-1-6-bisphosphate into D-glyceraldehyde-3-phosphate, which is produced from dihydroxyacetone phosphate and ATP. Glyceraldehyde-3-phosphate dehydrogenase (GAPA, GAPB, and GAPC) decreased in chloroplast of wheat seedlings (Kamal et al., 2012a). Glyceraldehyde-3-phosphate dehydrogenase (GAPDH) might be involved to abiotic stress by the glycolysis in plant (Hancock et al., 2005). These results are consistent with barley and rice under high-salt stress, whereas GAPDH decreased (Ueda et al., 2006). Sucrose synthase 4 is a membrane-associated form of the cytoplasmic enzymes that synthesize starch and involved in respiration and cell wall protein synthesis under salt stress in chloroplasts of wheat (Pang et al., 2010). Malate dehydrogenase (MDH) also displays a decrease in levels under salt stress in wheat (Kamal et al., 2012a). The chloroplastic NADP-dependent MDH is required for C4 photosynthesis to avoid the less-efficient photorespiration process in plants. NADP-MDH converts oxaloacetate to malate in the chloroplasts of mesophyll cells for its delivery to bundle sheath cells in C4 plants (Cushman, 1993).

ATPases are integral transport proteins that couple the hydrolysis of ATP and movement of protons across membranes to generate electrochemical gradients (Palmgren and Harper, 1999). The action of ATPases can influence salt stress mechanism by changing the membrane potential and proton gradient. The uptake, exclusion, and sequestration of Na^+^ and other ions are influenced by the above factors. V-type proton ATPase subunits, including VHA-B1, VHA-B2, and VHA-B3, were gradually decreased in chloroplast of wheat (Kamal et al., 2012a). NaCl helps to decrease the plasma membrane ATPases activity in both salt-sensitive and salt-tolerant wheat and tomato (Mansour et al., 2003). Cytoplasmic proteins might have important role for improving the unfavorable effects of high Na^+^ concentrations by the binding of ions. These changes in pump activities in response to salt exposure are not necessarily adaptive, but may be physiological consequences of salt stress, which causes Na^+^ ions to interfere with membrane integrity and alter protein activities (Mansour et al., 2003).

The abundance of 3-isopropylmalate dehydrogenase, which is involved in leucine biosynthesis, decreased in wheat chloroplasts after 1 day of salt-stress treatment (Kamal et al., 2012a). In contrast, glutamate dehydrogenase 1 and glutamine synthetase are increased. Glutamine synthetase is associated with the assimilation of enzyme for ammonia, and glutamate dehydrogenases 1 make bridge between carbon and nitrogen metabolism through the amination of 2-oxoglutarate to form glutamate (biosynthetic reaction) or the deamination of glutamate, generating ammonium and 2-oxoglutarate (catabolic reaction). Glutamate dehydrogenase 1 and glutamine synthetase along with other enzymes play vital roles in sustaining the balance between carbon and nitrogen assimilation (Miflin and Habash, 2002). Fumarate hydratase and isocitrate dehydrogenase in the citrate cycle were also unevenly changed in wheat (Kamal et al., 2012a). Isocitrate dehydrogenase supplies NADPH for plant defenses against oxidative stress (Millar et al., 2001) and generates 2-oxoglutarate, which is consumed in the glutamine synthetase–glutamate synthase cycle in carbon-limiting conditions that are experienced by plants under salinity stress (Hodges et al., 2003).

Germin-like protein was dynamically changed in wheat chloroplasts, but had increased at later stage under salt stress (Kamal et al., 2012a). Germin-like proteins are localized in the apoplast that are slackly allied with the cell wall matrix. Germin-like proteins are not only involved in germination, but also in biotic or abiotic stress in plants (Soussi et al., 1998; Schafleitner and Wilhelm, 2002). Germin like proteins has also been associated with SOD activity and shown oxalate oxidase activity, which is often increased by the stress exposure through the production of free-radicals. (Woo et al., 2000). In addition, decreased abundances of photosynthesis-responsive proteins might be the consequence of continuous and highly elevated Na^+^ ion uptake in the leaf and transpiration stream due to the reduced photosynthetic competence of plants under salt stress (Abbasi and Komatsu, 2004). The 3-oxoacyl-[acyl-carrier-protein] synthase I protein are involved in fatty-acid biosynthesis and was found to be unevenly changed under salt stress in wheat (Kamal et al., 2012a). This protein catalyzes the organic reaction of fatty-acid synthesis by the combining of two carbon acyl acceptor groups from malonyl-acyl carrier protein, resulting the produce the fatty acids from C-10 to C-16 and C-18 (Siggaard-Andersen et al., 1991). Alpha-1, 4-glucan-protein synthase [UDP-forming] is an ancestor of glycogen biosynthesis that is instigated by glycogen initiator synthase to catalyze the transfer of glucose from UDP-glucose to an acceptor protein, which was increased in chloroplast of wheat (Kamal et al., 2013b).

Carbonic anhydrase also was increased in wheat in response to salt treatment. This enzyme has important functions in assisting the transport of carbon dioxide and protons within the intracellular space, across biological membranes, and into the layers of the extracellular space. Besides, carbonic anhydrase is associated with respiration and photosynthesis in eukaryotes by catalyzing the rapid inter-conversion of carbon dioxide and water into carbonic acid, protons, and bicarbonate ions. The switch of bicarbonate to carbon dioxide facilitates its transport into cells, whereas the alteration to bicarbonate assists to trap carbon dioxide inside the cell (Hacisalihoglu et al., 2003).

Several pyridoxal phosphate-binding proteins, including cysteine synthase, PDX1.2, and PDX1.3, were shown to be differentially expressed in wheat chloroplasts in response to salt stress decreased at 2 d while increased at 3 d. These results suggest that pyridoxal phosphate-binding proteins respond to salt stress by inducing cysteine biosynthesis as a protective measure against high ion concentrations (Youssefian et al., 1993). Ferredoxin–NADP reductase and NAD(P)H–quinone oxidoreductase were also induced in salt-stressed wheat chloroplasts, a response that was also reported in tomato leaves (Zhou and Sauve, 2009). S-adenosylmethionine synthase and jasmonate O-methyltransferase generate methyl jasmonate by the methylation of jasmonate that are acted as cellular valve of various developmental courses and defense retorts in *Arabidopsis* (Seo et al., 2001).

The chloroplast translation elongation factor (EF)-Tu was increased in salt-stressed wheat plants. EF-Tu is a 46-kDa protein that actively binds and transports appropriate codon-specific aminoacyl-tRNAs to the aminoacyl site of the ribosome. EF-Tu from *Escherichia coli* interacts with unfolded and denatured proteins in a similar manner as molecular chaperones (Caldas et al., 1998). Uroporphyrinogen decarboxylase was increased, as has been observed in rice (Mock and Grimm, 1997). However, this enzyme, which is thought to be a cell death-responsive protein (Zang and Komatsu, 2007), was decreased in the chloroplasts of salt-stressed wheat. Stress-associated protein 7, which contains zinc finger A20 and AN1 domains, was increased in wheat chloroplasts during stress. This pattern was also found in rice and *Arabidopsis* (Vij and Tyagi, 2006).

The chloroplast protein nudix hydrolase 20, which belongs to a family of proteins that catalyze the hydrolysis of nucleoside diphosphates, exhibits fluctuating levels during stress periods in wheat (Kamal et al., 2012a). In *Arabidopsis*, levels of nudix hydrolase increase following oxidative stress (Jambunathan and Mahalingam, 2006). Although this protein was originally proposed to have housekeeping-functions such as RNA processing, valve of calcium channel, and directive of ERK signaling (McLennan, 2006). Serine/threonine protein kinase was increased in chloroplast of wheat that has also been connected to biotic stress as well as dehydration (Chinnusamy et al., 2004). This kinase might have important functions in abiotic and biotic stress-signaling pathways. Supporting this speculation, SnRK family (SNF-1 related protein kinases) were also increased or activated under osmotic stress (Veeranagamallaiah et al., 2008).

In Table 1, separation of wheat (cv. Zhengmai 9023) leaf proteins by 2-DE followed by their identification using Q-TOF MS has revealed that salt-responsive proteins are mainly involved in membrane transport, ROS detoxification, ATP synthesis, carbon metabolism, and protein folding (Gao et al., 2011). The higher abundances of H^+^-ATPases, glutathione S-transferase, ferritin, and triosephosphate isomerase might be responses salt tolerance mechanism at the basis of molecular levels in Zhengmai 9023. Biotic and abiotic stresses often lead to the production of excess amounts of ROS (singlet oxygen, superoxide radical, hydroxyl radical, and hydrogen peroxide) that are protecting the oxidative damage. Hence, ROS (antioxidants and antioxidant enzymes) are associated to suspend the cascades of abandoned oxidation in cell organelles (Shigeoka et al., 2002).

Glutathione S-transferases (GSTs) participated in various cellular metabolisms including scavenging the ROS. GST has been draw attention in stress responses such as pathogen attack, oxidative stress, heavy-metal toxicity, salt stress. Ferritin protein were also increased significantly under salt stress in wheat (Jiang et al., 2007), *Arabidopsis* (Ndimba et al., 2005), and rice (Parker et al., 2006). It is worked as iron-reserve proteins and plays a defending responsibility against the lethal effects of iron in cells. Ferrous iron and H_2_O_2_ produce the hydroxyl radicals through Fenton reactions that are most hazardous type of ROS in plant cells that are potentially neutralized by the ferritin. Triosephosphate isomerase generates the reversible alteration of dihydroxyacetone phosphate to glyceraldehyde-3-phosphate and is involved in many metabolic pathways, including glycolysis, the Calvin cycle, and glycerol metabolism (Kamal et al., 2015). Glucose catabolism might be amplified under salt stress in wheat for supplementary energy for the detoxification and repair of damage caused by oxidative molecules (Gao et al., 2011).

### Changes in wheat proteome composition under drought stress

Drought resulting from a shortage of water induces osmotic stress, which is a major limiting factor for plant growth, development and quality crop production (Mohammadi et al., 2012). Osmotic stress causes a reduction in CO_2_ fixation, thereby decreasing NADP^+^ regeneration by the Calvin cycle. As a consequence, the photosynthetic electron transport chain becomes over-reduced, leading to the formation of excess ROS, predominantly superoxide radicals and singlet oxygen, which impair the function of chloroplast proteins involved in photosynthesis (Ashraf and Harris, 2013). The mechanisms involved in the drought response have been extensively studied at the protein level using proteomics over the last few decades. However, only a few researcher studies on chloroplast of wheat proteome under drought stress (Kamal et al., 2013a). Redox regulation, oxidative stress response, signal transduction, protein folding, secondary metabolism, and photosynthesis related proteins were respond under drought stress in chloroplast of wheat. The levels of metabolism-related proteins were increased under polyethylene glycol-treated and drought-stressed in leaves of wheat seedlings, whereas the levels of proteins related to energy production and protein synthesis were decreased. Notably, the root was initiated to be the mainly drought-responsive organ, as it displayed the largest changes in protein abundance in response to drought stress (Mohammadi et al., 2012).

Proteins involved in carbon metabolism generally demonstrate marked changes in response to water deficit (Figure 2). Starch biosynthesis and accumulation in endosperm cells primarily occur during the grain filling stage. Among starch-synthesizing enzymes, ADP-glucose pyrophosphorylase is considered to be the rate-limiting enzyme, as it generates the sugar nucleotide ADP-glucose and inorganic pyrophosphate from glucose-1-phosphate and ATP as the first step in the synthetic process (Dai, 2010). In Table 1, wheat grain proteome analysis by linear and non-linear 2-DE and MALDI-TOF MS revealed that both the small and large subunits of ADP-glucose pyrophosphorylase, in addition to ascorbate peroxidase (APX) and G beta-like protein, were down-regulated in drought-sensitive variety Janz, whereas the level of the enzymes in the tolerant variety Kauz did not show any significant changes in response to drought stress (Jiang et al., 2012). In contrast, CAT isozyme 1, WD40 repeat protein, late-embryogenesis subunit (LEA), and alpha-amylase inhibitors displayed higher abundances in Kauz, but were down-regulated or unchanged in Janz under drought conditions. Higher abundance of sucrose synthase together with unaltered levels of ADP-glucose pyrophosphorylase results in higher starch synthesis even under drought conditions. This finding indicates that these enzymes are strongly associated with the higher drought resistance of the Kauz variety of wheat (Jiang et al., 2012).

RuBisCO, the most abundant leaf protein plays a major role in CO_2_ fixation and photorespiration in C_3_ plants (Jensen and Bahr, 1977). It constitutes a large pool of stored leaf nitrogen (15–30%) that is rapidly remobilized under stress and senescence (Feller et al., 2008). The activity of RuBisCO is regulated by RuBisCO activase protein, which removes the tightly bound sugar-phosphates from the active centers of RuBisCO, leading to its reactivation (Gutteridge and Gatenby, 1995; Spreitzer and Savucci, 2002). It is considered that the impaired activity of RuBisCO under drought condition may be associated with the reduction in ATP concentration (Tezara et al., 1999). RuBisCO activase is sensitive to the high temperature that is often associated with drought (Crafts-Brandner and Salvucci, 2000).

Drought has been shown to induce metabolic impairment such as decrease in the RuBisCO activity (Tezara et al., 2002; Bota et al., 2004). This declined activity is associated with low stomatal conductance and chloroplast CO_2_ concentration and is not induced by decreased relative water content under water stress (Flexas et al., 2006). The effects of drought stress on the amount of wheat RuBisCO protein differ among reports, from slightly enhanced to no change (Demirevska et al., 2008). The RuBisCO level also found to be increased during leaf expansion and reached the highest after full leaf expansion (Ishida et al., 1997). However, during leaf senescence, RuBisCO is degraded and its nitrogen is re-mobilized and translocated into growing organs and used for their growth. Studies have indicated that a majority of RuBisCO are degraded within chloroplasts as RuBisCO levels are rapidly decreased in conjunction with the loss of chloroplasts in barley and wheat leaves during senescence (Wardley et al., 1984). Interestingly, magnitude of the decrease in RuBisCO content during senescence was found to be enhanced by exogenous abscisic acid (ABA) in rice leaves (Fukayama et al., 2010). The SDS-dependent proteases induced by ABA might be responsible for senescence related degradation of chloroplast proteins including RuBisCO and RuBisCO activase. Notably, in wheat leaves, both drought and exogenous ABA pretreatment resulted in increased expression of RuBisCO activase (Asghari and Ebrahimzadeh, 2006). A separate study on proteome changes in wild and modern wheat leaves upon exposure to drought also revealed elevated level of RuBisCO both at protein and transcript levels (Budak et al., 2013). When separated on 2D gels, several subunits of RuBisCO are located in different gel areas, and likely represent different splice variants, post-translationally modified isoforms, or cleaved isoforms of the same protein (Weiss and Gorg, 2007). The RuBisCO subunits are unevenly expressed in the chloroplasts of drought-stressed wheat seedlings (Kamal et al., 2013a). Isoforms of RuBisCO activase might have significant roles in alleviating and scheming proteolysis (Schwartz et al., 1995) and in maintaining chloroplast functioning during drought stress (Huo et al., 2004).

H^+^-transporting two-sector ATPase and membrane-bound ATP synthase subunit b were localized in plasma membranes that are dynamically changed under drought stress in chloroplast of wheat (Kamal et al., 2013a). Non-phosphorylated ATPase is implicated in ion transportation. F-type enzymes in the inner mitochondrial and thylakoid membranes also function in ATP synthesis. Additionally, V- and A-type enzymes contained analogous structure, whereas pump H^+^ ions are not involved in the synthesis of ATP (Flexas et al., 2006). A number of integral membrane proteins, include members of the chloride carrier/channel (ClC) family, were also increased in wheat exposed to drought stress. The members of the ClC family are response to voltage-regulated in ion channels that provide a range of physiological responsibilities including cell volume regulation, membrane potential stabilization, signal transduction, and trans-epithelial transport (Worden et al., 2009; Ford et al., 2011). ClC family in plants might have function as anion channels for nitrate homeostasis. Analysis using the WolF PSORT sub-cellular location predictor indicates that CLC proteins have 14% similarity with phytoene synthase proteins (Kamal et al., 2013a), which are located in chloroplasts (Römer et al., 1993).

Cytochrome b6-f complex is an enzyme located in the thylakoid membrane that is intervened electron transfer between photo-systems (PS) I and II including cyclic electron flow around PSI and state transitions (Hurt and Hauska, 1981). In wheat plants exposed to drought, the iron-sulfur subunit (petC) of the cytochrome b6-f complex was increased after 3 days of treatment, as has been observed in drought-stressed rice (Ali and Komatsu, 2006). However, petC was decreased after 6 and 9 days of drought stress, which leads to photosynthesis degradation and alters chloroplast structure, as well as promoting leaf senescence (Kamal et al., 2013a). In contrast, the expression of chloroplast OEE1 was increased after 9 days of drought stress. Murota et al. (1994) reviewed the function of OEE1in salt adaptation in photo-autotrophically cultured green tobacco cells, mangrove, and rice (Sugihara et al., 2000).

Adenylate kinase (ADK) is a small ubiquitous enzyme involved in the metabolism of purine nucleotides and is essential for cell maintenance and growth. In drought-stressed wheat, ADK levels were increased after 3 days, but had decreased after 6 and 9 days, possibly due to cell death (Kamal et al., 2013a). As ADK participates in ATP biosynthesis, drought-tolerant genotypes may provide more ATP for maintaining cellular activities under drought stress (Gong et al., 2010). Although plastid stromal ADK plays important roles in the coordination of metabolism and growth, the importance of this isoform is suggested to be tissue-dependent (Carrari et al., 2005). ADK is a key enzyme in energy metabolism, as it catalyzes a reversible transphosphorylation reaction that converts ADP to ATP and AMP, and is critical for many processes in living cells (Pradet and Raymond, 1983).

In Table 1, a shotgun proteomic approach for investigating quantitative changes in protein abundance of two drought-tolerant and one intolerant cultivar of Australian bread wheat revealed a higher abundance of CAT and three isoforms of SOD, namely chloroplastic and cytosolic Cu/Zn-SOD, and mitochondrial Mn-SOD, in response to water deficit stress (Ford et al., 2011). Moreover, a coordinated stress-induced down regulation of proteins involved in photosynthesis and the Calvin cycle was observed, irrespective of cultivar, suggesting this may be a defense strategy to avoid excess ROS generation (Figure 2).

ABA-responsive marker proteins, such as LEA proteins and PP2C family phosphatases, are strongly increased in different plant species in response to ABA and abiotic stresses such as cold and drought and constitute the core proteins of ABA signaling pathways (Vaseva et al., 2010). A total of six LEA proteins, ABA-responsive proteins, and protein phosphatases were identified as ABA-responsive proteins in wheat roots under drought stress (Alvarez et al., 2014). In addition, numerous proteins involved in secondary metabolism, including those related to jasmonic acid, lignin, oxylipin, and phenylpropanoid metabolic processes, are increased in response to ABA treatment in wheat roots under drought stress, (Alvarez et al., 2014).

Drought and high-temperature stress adversely affect wheat seed yields and quality, particularly the composition of seed storage proteins that form during the grain filling stage. Water stress during the grain development stage markedly affects the levels and composition of grain carbohydrates, and storage protein synthesis and accumulation, resulting in poor grain quality (Jiang et al., 2007). However, the content of albumin and gliadin are increased in grains in response to drought, whereas globulin and glutenin do not markedly change (Zhang et al., 2013). Similarly, several α-gliadins, γ-gliadins, and low-molecular-weight glutenins increased in wheat grains exposed to high temperature, whereas α/β-gliadin, ω-gliadin, and globulins decreased (Yang et al., 2011). 1-Cys peroxiredoxin was differentially regulated under drought and high-temperature stress. In wheat exposed to high temperature and drought stress, differentially changed proteins were predominantly involved in stress/defense, signaling pathways, redox regulation, and energy metabolism (Yang et al., 2011). High-temperature stress often increases stomatal conductance, respiration, leaf transpiration, and oxidative stress (Rizhsky et al., 2002), shortens the duration of grain filling, and enhances gluten protein accumulation and starch synthesis (Hurkman et al., 2009). Plants respond to heat by signaling via ABA, ethylene and salicylic acid, scavenging of ROS via the production of antioxidants, and transcriptional activation of stress-related proteins (Wahid et al., 2007). A number of proteins, including heat shock, carbohydrate metabolism, and storage proteins, were changed in the wheat proteome in response to high temperature. Albumin proteins involved in primary metabolism were not changed significantly under high temperature (Yang et al., 2011).

## Responses of wheat to heavy metal stresses

### Responses of wheat to aluminum stress

Aluminum (Al) toxicity is a major constraint for agricultural crop production on acid soils, which are estimated to comprise over 50% of the world's potentially arable lands, (Yang et al., 2007). Among the various Al toxicity symptoms, the most sensitive response is the inhibition of root elongation. Toxic levels of Al in acid soils hinder root growth and cause a significant reduction in yields of Al-sensitive crops (Dechassa et al., 2011). In addition, Al stress leads to a number of cellular, physiological, and biochemical disorders (Kochian, 1995; Yamamoto et al., 2002; Suping et al., 2009). Based on the assessment off morphological and physiological features, root lengths and weights were shown to be severely reduced by Al stress (150 uM) in wheat, and the Al ion concentration within tissues was significantly increased (Oh et al., 2014). In Table 1, in proteomics-based experiments examining the effects of Al stress on root proteins of wheat, 47 differentially change proteins were identified by LTQ-FTICR MS after separation by 2-DE. Of these proteins, 19 proteins were significantly increased, whereas 28 proteins were decreased (Oh et al., 2014).

β-amylases are exoamylases that release maltose from the non-reducing ends of glucans or dextrins by cleavage of α(1–4) linkages. Although plant β-amylase is considered to be a key enzyme for catalyzing the breakdown of starch, the enzyme was shown to be located outside the plastids, and its role in starch breakdown remains unclear (Bancel et al., 2010). S-adenosylmethionine synthetase is related to the ethylene-mediated inhibition of root growth and may also be involved in the alteration of cell wall structures and polymers in the roots of wheat under Al stress (Fukuda et al., 2007). Oxalate oxidase catalyzes the oxidation of oxalic acid to CO_2_, Ca^2+^, and H_2_O_2_, and is stored as insoluble calcium salt upon reaction with molecular oxygen (Lane et al., 1993). The Al–induced activation of oxalate oxidase in wheat roots may be involved in detoxifying H_2_O_2_ (Delisle et al., 2001). When exposed to Al, tolerant plants secrete organic acid anions to chelate and immobilize Al^3+^ at the root surface, thereby preventing the excess accumulation of Al^3+^ in the root system (Kobayashi et al., 2005, 2007). Malate dehydrogenase (MDH) was increased in the roots of wheat under Al stress. MDH catalyzes a reversible reaction that forms malate and NAD from oxaloacetate and NADH, and is therefore a key enzyme in the TCA cycles of prokaryotes and eukaryotes (Ding and Ma, 2004).

Cysteine synthase is a key enzyme for mediating Al tolerance and is increased in the roots of wheat exposed to Al. Cysteine synthase is required for cysteine biosynthesis in plants and is one of the most important enzymes in sulfur assimilation leading to the production of antioxidants and metal chelators, such as glutathione, metallothionein, and phytochelatin (Yang et al., 2007). Triosephosphate isomerase is increased in wheat roots under Al stress, whereas glyceraldehyde-3-phosphate dehydrogenase is decreased in roots (Figure 2). Glyceraldehyde-3-phosphate dehydrogenase catalyzes the conversion of glyceraldehyde-3-phosphate into 1,3-bisphosphoglycerate, which is then converted to 3-phosphoglycerate by the action of phosphoglycerate kinase, which is an important transferase enzyme in glycolysis pathway and is also increased in wheat roots under Al stress (Watson et al., 1982; Zhou et al., 2009). Chloroplast phosphoglycerate kinase is encoded as a polyprotein precursor that is comprised of at least four subunits, which have been separated and shown to be conserved tetrapeptides (Nowitzki et al., 2004).

The activity of APX is increased in response to Al stress and to other abiotic stresses such as salinity, chilling, metal toxicity, drought, and heat. APX is reported to have a possible role in detoxifying H_2_O_2_ in the cells of various plant species (Davis and Swanson, 2001; Bueno and Piqueras, 2002). Quinone reductase was differentially changed under AL stress in wheat (Oh et al., 2014). It is a homodimeric FAD-containing enzyme that catalyzes the obligatory NAD(P)H-dependent two-electron reduction of quinones to protect cells against the toxic and neoplastic effects of free radicals and ROS arising from one-electron reductions in tomato under Al stress (Zhou et al., 2009). Methionine synthase was changed under Al stress in leaves of wheat (Oh et al., 2014). It catalyzes the transfer of a methyl group from methyltetrahydrofolate to homocysteine, generating tetrahydrofolate and methionine, which is converted to adenosylmethionine (AdoMet), which serves as a methyl donor in numerous biosynthetic reactions (Matthews et al., 1998). Elongation factor 1-gamma was changed under Al stress in leaves of wheat (Oh et al., 2014) that is involved in translational control by linking the alpha and beta subunits of eukaryotic elongation factor during the GDP to GTP exchange reaction (Thornton et al., 2003). Succinyl CoA ligase, which catalyzes the reversible conversion of succinyl-CoA to succinate, is increased in the roots of wheat under Al stress (Drummond et al., 2001). The formation of a nucleoside triphosphate molecule from an inorganic phosphate molecule and a nucleoside diphosphate molecule were facilitated by this enzyme. Succinyl CoA ligase is also a key catalyst in the citric acid cycle, which occurs in the matrix of mitochondria (Chakrabarty, 1998; Oh et al., 2014).

Plant annexins form a multigene family, are responsive to drought, salt, and cold stresses in *Arabidopsis* (Cantero et al., 2006). Different isoforms of annexin are reported to possess different enzyme or other protein activities, including phosphodiesterase, peroxidase, F-actin binding, and calcium channel activities. Plant annexins may also participate in the regulation of callose and cellulose synthase activity. Annexins have been implicated in wheat under cold stress (Breton et al., 2002). UDP-d-glucuronate decarboxylase catalyzes the formation of UDP-D-xylose from UDP-D-glucuronate in an irreversible reaction with the help of UDP-D-glucuronate decarboxylase (Zhang et al., 2005). Fructose-bisphosphate aldolase is increased in wheat roots under Al stress (Oh et al., 2014) that are catalyzes the cleavage of fructose 1-6-bisphosphate into the glycolytic intermediates D-glyceraldehyde-3-phosphate and dihydroxyacetone phosphate (Johnson et al., 1989). This enzyme might be facilitated the acclimation to Al through the synthesis of ATP in glycolysis and ethanolic fermentation (Andrews et al., 1994).

### Responses of wheat to Cadmium and copper stresses

Cadmium (Cd) is a non-essential element that is highly toxic to plants, even at very low concentrations. Plants readily absorb Cd from the soil through membrane-bound cation transporters, which are primarily responsible for the uptake of essential trace elements (Welch and Norvell, 1999). Within cells, Cd^2+^ disturbs normal cellular functions by displacing Zn^2+^, Ca^2+^, and Fe^2+^ from proteins, leading to enzyme inactivation and the disruption of vital biochemical pathways related to sugar metabolism, nitrate absorption, and photosynthesis. Plants suffering from Cd toxicity exhibit leaf chlorosis, wilting, growth inhibition, and cell death (Vassilev et al., 1995; Sanità di Toppi and Gabbrielli, 1999; Clemens, 2006). Morphological and physiological analyses of wheat exposed to high Cd concentrations showed that leaf elongation was decreased, and that H_2_O_2_ and malondialdehyde (substrate of lipid peroxidation) levels significantly increased (Wang et al., 2011). Further, 2-DE analysis led to the identification of proteins that were differentially changed in response to Cd stress. A number of oxidative stress-related proteins such as APX, glutathione-S-transferase (GST), and SOD are increased in response to Cd accumulation (Figure 2, Table 1). GST plays a role in the oxidative stress response by activating GSH-dependent peroxidases, which is able to detoxify metabolites arising from oxidative damage (Edwards et al., 2000). Additionally, GST is thought to be directly involved in heavy metal detoxification in plant cells by forming complexes with metal ions, such as the GSH-Cd complex (Adamis et al., 2004; Mishra et al., 2009). The increases in APX, SOD, and GST in response to Cd stress might play an important role antioxidant defense.

H^+^-transporting two-sector ATPase alpha and beta chain was increased by Cd and 1,2,4- Trichlorobenzene (TCB) stresses in leaves of wheat, which is essential to maintain the ATP high level required by the stressed cells. The vacuolar proton-ATPase (V-ATPase) subunit A was increased by Cd and TCB in wheat (Ge et al., 2009) that are plays an important role in plant responses to environmental stresses (Fukuda et al., 2004). V-ATPase uses the energy derived from the hydrolysis ATP to establish the electrochemical gradient of H^+^ across vacuolar membrane (Sun-Wada et al., 2003), which is also the driving force for the accumulation of toxic ions and other solutes in the vacuole (Hamilton et al., 2001). ACC synthase was increased in leaves of wheat by the accumulation of Cd and TCB (Ge et al., 2009). The induction of ACC synthase can stimulate ethylene production (Wong et al., 2001). Ethylene, as an intermediary of the stressful signal, plays an key defense role such as regulating plant growth and development, inducing the expression of various defensive genes in response to Cd and TCB stresses (O'Donnell et al., 1996).

Copper (Cu) is one of the most hazardous pollutants in agricultural fields. Environmental levels of Cu are rapidly increasing due to excessive use of phosphate fertilizer and irrigation with sewage and water polluted by activities of the metallurgy industry. Cu not only reduces crop yields, but also accumulates in the edible parts of plants, allowing it to easily enter the food chain and cause adverse effects on human health (Cobbett and Goldsbrough, 2002). The results of morphological and physiological studies have demonstrated that plant height, root length, fresh and dry weight, and pigment content of leaves (carotenoid and chlorophyll) are significantly decreased during stress. In addition, the malondialdehyde (MDA) content of roots and leaves increases significantly while Cu contents increased gradually, whereas they having a strong correlation during Cu stress (Li et al., 2013). 2-DE analysis of wheat proteins under copper stress led to the identification of 93 proteins, which were involved in signal transduction, carbohydrate metabolism, protein metabolism, energy production, and transportation, in roots and leaves. Moreover, a number of proteins related to carbohydrate metabolism, protein metabolism, and photosynthesis were specifically changed in the roots or leaves, and included lipoxygenase 2.1 in the leaves and 5, 10- methylenetetrahydrofolate reductase in the roots (Li et al., 2013).

## Prospects and challenges

For plants to respond optimally to the ever-changing environment, precise modulation of the proteome is essential for adaptive responses in metabolism and development. Plant adaptive responses to stress need to be decoded in terms of the specific, underlying biological processes. A proteomic approach is useful not only for reconstruction of the plant response to various stresses as a whole, but is also to able to allow different stress responsive pathways to be individually dissected. Most wheat proteomic studies published to date have mainly focused on the effects and plant responses to individual stresses, and proteomic changes in response to multiple stresses remain to be elucidated. However, such an approach would allow a greater understanding of the cross-talk that occurs between different stress signal pathways.

Conventional gel-based proteomic approaches, and gel free-mass spectrometry (MS)-based methods involving label-based and label-free protein quantification have been extensively used with their own advantages and disadvantages for characterization of stress-responsive proteins in wheat (Kong et al., 2010; Oh et al., 2014; Zhang et al., 2014). Gel based approaches are widely used for their simplicity, reproducibility, wide molecular weight coverage, and detection of post-translational modifications (Ghosh and Xu, 2014). Nonetheless, 2-DE approach has some known limitations like issues related to reproducibility, identifying low-abundance and hydrophobic proteins, exceedingly large or small proteins, as well as basic proteins and co-migration of proteins (Hossain et al., 2013). However, for investigation of membrane proteins interactions, blue native PAGE (BN-PAGE), a gel-based approach has become a method of choice for its simplicity and suitability for lipophilic entities. A systematic strategy involving blue native polyacrylamide gel electrophoresis (BN-PAGE) and SDS-PAGE has been designed for the proteomic analysis of wheat chloroplast protein complexes (Meng et al., 2011). Using this method, chloroplast envelopes, thylakoids, and stromata were enriched effectively, and more than 18 complexes were obtained by BN-PAGE.

The inherent limitations of 2-DE can be overcome by the multidimensional protein identification technique (MudPIT), a non-gel method of shotgun proteomics approach. Quantification in MudPIT analysis is usually performed through *in vitro* labeling techniques such as isotope tags for relative and absolute quantitation (iTRAQ) and isotope coupled affinity tags (ICAT). Exploiting the ICAT labeling method, Islam et al. (2003) successfully unraveled the expression of wheat seed proteins, which were reported to be difficult through 2-DE approach due to co-synthesis of proteins by genes belong to three different genomes namely, A, B, and D. Moreover, metabolic seed protein fraction in durum wheat were analyzed through gel-free shotgun proteomics, that allowed identifying over 380 proteins exhibiting greater molecular weight diversity ranging from 7 to 258 kDa (Fercha et al., 2013).

The accuracy of proteomic data is highly dependent on the sample preparation and analysis techniques, including the isolation, separation, visualization, and identification of the full complement of proteins. Despite recent advances, more emphasis needs to be given to protein extraction protocols, particularly for very low abundance proteins such as transcription factors, kinases, and transporters. Fluorophore-tagged protein immune-precipitation and label-free MS-based quantification techniques have been shown to be superior to the classical 2-DE-based proteomic approach for the accurate identification of low-abundance signaling and regulatory protein complexes (Smaczniak et al., 2012). In addition, advanced tools such as laser-capture micro-dissection (Dembinsky et al., 2007) for tissue proteomics could be adapted for the identification of tissue- and cell-specific proteins involved in plant stress tolerance mechanisms. Moreover, the selected reaction monitoring technique is an emerging MS technique for the targeted and highly sensitive identification and accurate quantitation of very low abundance proteins and modified peptides in complex biological mixtures (Calvo et al., 2011; Picotti and Aebersold, 2012).

The development of effective strategies for post-proteomics or functional proteomics analyses requires an accurate understanding of the effects of post-translational modifications on individual proteins and the interactions between modified proteins and their associated metabolic pathways, in addition to how these pathways are integrated into the cellular metabolic network. In particular, the post-translational protein modifications such as phosphorylation, glycosylation, and oxidation, which are thought to play key roles in stress signaling, require greater understanding at the molecular level. MS-based phosphoproteomic technology has become an invaluable tool for the identification of phosphoproteins and mapping of phosphorylation sites. Nevertheless, identification of the *in-vivo* protein phosphorylation sites, which is required for the functional characterization of target proteins, is a difficult challenge for any phosphoproteomic study. Immobilized metal affinity chromatography (IMAC) and immunoprecipitation using antibodies against phosphorylated amino acids are the two most common pre-fractionation techniques used in MS analysis (Zhang et al., 2014). Significant recent progress has been made in the quantitative and dynamic analyses of mapped phosphorylation sites. The isolation of phosphopeptides by IMAC followed by MS/MS or MS(n) analysis has enabled the detection of hundreds of new *in-vivo* phosphorylation sites (Bentem et al., 2006). The more advanced iTRAQ and stable isotope labeling by/with amino acids (SILAC) labeling techniques label peptides *in vitro* just prior to MS or label proteins during cell growth, respectively, and allow for changes in individual phosphorylation sites to be measured over time in response to stress.

In addition to the development of functional proteomics, a greater focus on subcellular proteomics of wheat is warranted, as organelle proteome studies generate detailed information on the intrinsic mechanisms of plant abiotic stress responses. Isolation of the target organelle from total tissue extracts is one of the most challenging aspects of subcellular proteomics. Conventional methods of subcellular fractionation typically involve differential and density-gradient centrifugation, which use a series of centrifugation steps to separate different cellular compartments or organelles from cell homogenates based on mass and/or density. In contrast, free-flow electrophoresis fractionates organelles based on their net global isoelectric charges or electrophoretic mobilities. Moreover, immunoaffinity purification is a more advanced technique that has been used successfully for the isolation of organelles with high specificity and yields (Ackermann and Berna, 2007). Despite the application of these advanced proteomics techniques, many organelle proteins, including both stress-induced and house-keeping proteins, remain unclassified. Future initiatives aimed at identifying and characterizing organellar proteins are expected to aid in the global proteomic profiling of environmentally stressed plants.

The combination of these proteomic approaches will facilitate characterization of target regulatory proteins and aid in their molecular cloning to address fundamental questions about plant physiology under abiotic stress. Finally, integration of wheat “Omics” data, such as genomics, transcriptomics, proteomics, metabolomics, and interactomics, with rapidly evolving bioinformatics tools and interactive databases is essential to construct mathematical models that will provide a more comprehensive picture about the mechanisms underlying plant performance under adverse conditions.

### Conflict of interest statement

The authors declare that the research was conducted in the absence of any commercial or financial relationships that could be construed as a potential conflict of interest.

## Abbreviations

2-DE: two-dimensional polyacrylamide gel electrophoresis
IEF: isoelectric focusing
pI: isoelectric point
IPG: immobilized pH gradient
iTRAQ: *isobaric tags for relative and absolute quantitation*
LC: liquid chromatography
MS: mass spectrometry
SILAC: stable isotope labeling by amino acids in cell culture
RuBisCO: ribulose 1,5-bisphosphate carboxylase/oxygenase
OEE: oxygen-evolving enhancer protein
ROS: reactive oxygen species
LEA: late-embryogenesis abundant
ABA: abscisic acid.
